# Effects of small heat shock proteins from thermotolerant bacteria on the stress resistance of *Escherichia coli* to temperature, pH, and hyperosmolarity

**DOI:** 10.1007/s00792-023-01326-y

**Published:** 2024-01-22

**Authors:** Yu Sato, Kenji Okano, Kohsuke Honda

**Affiliations:** 1 Division of Agricultural Sciences, Graduate School of Sciences and Technology for Innovation, Yamaguchi University, Yamaguchi, Japan; 2 Graduate School of Science and Technology for Innovation, Yamaguchi University, Yamaguchi, Yamaguchi 753-8515 Japan; 3 Research Center for Thermotolerant Microbial Resources, Yamaguchi University, Yamaguchi, 753-8515 Japan; 4 Department of Life Science and Biotechnology, Kansai University, Suita, Osaka 564-8680 Japan; 5 International Center for Biotechnology, Osaka University, 2-1 Yamada-oka, Suita, Osaka 565-0871 Japan; 6 Industrial Biotechnology Initiative Division, Institute for Open and Transdisciplinary Research Initiatives, Osaka University, 2-1 Yamada-oka, Suita, Osaka 565-0871 Japan

**Keywords:** Heat shock protein, Thermophiles, Gene expression, Stress resistant

## Abstract

Small heat shock proteins (HSPs), such as HSP20, represent cellular thermal resistance mechanisms, to avoid protein aggregation at elevated temperatures. Recombinantly expressed HSP20s serve as a molecular tool for improving the tolerance of living cells to various physical and chemical stressors. Here, we aimed to heterologously express 18 HSP20s from 12 thermotolerant bacteria in *Escherichia coli* and evaluate their effects on various physical and chemical cellular stresses. Seventeen HSP20s were successfully expressed as soluble proteins. Recombinant *E. coli* cells were subjected to heat, cold, acidic, alkaline, and hyperosmolar stress to evaluate the effects of HSP20 proteins on stress resistance. Notably, the overexpression of 15 HSP20s enhanced the stress resistance of *E. coli* compared to that of the control strain*.* In particular, HSPs from *Tepidimonas sediminis* and *Oceanithermus profundus* improved the stress tolerance of *E. coli* under all tested conditions. In addition, *E. coli* harboring HSP20 from *T. sediminis* retained cell viability even after heat treatment at 52 °C for 5 days. To our knowledge, this is the first report of *E. coli* tolerance to prolonged (> 100 h) high-temperature stress. These findings indicate the potential of thermotolerant HSPs as molecular tools for improving stress tolerance in *E. coli*.

**Supplementary Information:**

The online version contains supplementary material available at 10.1007/s00792-023-01326-y.

## Introduction

Living cells are equipped with various molecular machineries to adapt to physical and chemical stresses, such as cold, heat, acid, alkali, and salinity. Heat shock proteins (HSPs) are one such machinery which are found ubiquitously. On exposure to environmental stress, cells increase the expression of HSPs that function as molecular chaperones to prevent protein aggregation (Whitley et al. 1999). HSPs are classified into the following five major families: HSP100, HSP90, HSP70, HSP60, and small HSP. Among them, small HSPs are defined as those with molecular masses ranging from 12 to 43 kDa (Basha et al. 2012), with majority of the HSPs between 14 and 27 kDa (Narberhaus 2002).

Small HSPs are ATP-independent molecular chaperones which prevent protein aggregation in living cells (Bepperling et al. 2012). In particular, HSP20, a type of small HSP, has been well studied in eukaryotes, archaea, and bacteria, and plays crucial roles against many physical and chemical stresses (Tanguay and Hightower 2015). The transcription of small HSPs is induced by multiple stresses, such as heat, cold, starvation, pH changes, chemicals, biomodulators, and posttranslational modifications (e.g., Guo et al. 2020; Cong et al. 2020), to adopt these stresses. For instance, endogenous small HSP20s from *Escherichia coli* (ibpA and ibpB) contribute to heat and hydrogen peroxide resistance (Kitagawa et al. 2000). In addition, HSP20s encoded by a genomic island improved the cell viability of *E. coli* at 60 °C (Li and Gänzle 2016). Besides heat stress, the co-expression of HSP20, glutaredoxin-3, iron-binding protein, and 2Fe-2S ferredoxin in *Deinococcus radiodurans* enhances its resistance to hydrogen peroxide (Singh et al. 2014). HSP20 is also essential for desiccation tolerance in *Azotobacter vinelandii* (Cocotl-Yañez et al. 2014). HSP20s are also found in several types of bacteriophages (Maaroufi and Tanguay 2013), and are presumably involved in the maturation of capsid proteins and/or stress resistance in their hosts (Sullivan et al. 2010; Chen et al. 2020). Furthermore, heterologous overexpression of HSP20 derived from multiple organisms can enhance cellular tolerance to diverse stresses in *E. coli* cells (Table 1).

**Table 1:** Previous studies for small HSPs on stress resistance of *Escherichia coli*

Name	Gene source	Domain	Host strain	Vector	Tested stress	References
MpHsp17.6	*Methanolobus psychrophilus*	Archaea	BL21 (DE3)	pET28a	Oxidation	Ma et al. (2021)
Pfu-sHsp	*Pyrococcus furiosus*	Archaea	BL21 (DE3)	pET19b	Heat (50 °C)	Laksanalamai et al. (2001)
Saci_Hsp20	*Sulfolobus acidocaldarius*	Archaea	BL21 (DE3)	pET28a	Heat (50 °C)	Roy et al. (2018)
SsHsp14.1	*Sulfolobus solfataricus*	Archaea	BL21 (DE3)	pET28a	Heat (55 °C)	Wang et al. (2010)
S.so-Hsp20	*Sulfolobus solfataricus*	Archaea	BL21 (DE3)	pET28a	Heat (50 °C), cold (4 °C)	Li et al. (2012)
tpv-Hsp14.3	*Thermoplasma volcanium*	Archaea	BL21 (DE3) pLysS	pDrive	Heat (52 °C)	Kocabıyık and Aygar (2012)
Al-IbpA	*Acholeplasma laidlawii*	Bacteria	BL21 (DE3)	pET15b	Heat shock (46 °C)	Kayumov et al. (2017)
DR1114	*Deinococcus radiodurans*	Bacteria	EPI300	pASK-IBA3	Oxidation	Singh et al. (2014)
MTB_Hsp16.3	*Mycobacterium tuberculosis*	Bacteria	BL21 (DE3)	pET-20b( +)	Heat (48 °C)	Valdez et al. (2002)
Oo-Hsp20	*Oenococcus oeni*	Bacteria	BL21 (DE3)	pTriEx1.1	Heat (52 °C), salinity, pH, oxidation	Qi et al. (2020)
Af-Hsp26	*Artemia franciscana*	Eukaryote	BL21 (DE3)	pET21( +)	Heat (54 °C)	Crack et al. (2002)
Br-Hsp20	*Brachionus sp.*	Eukaryote	BL21 (DE3) pLysS	pET100/D-TOPO	Heat (54 °C), oxidation	Rhee et al. (2011)
CsHsp17.5	*Castanea sativa*	Eukaryote	BL21 (DE3)	pRSET	Heat (50 °C) and cold (4 °C)	Soto et al. (1999)
CgHsp22.4	*Chaetomium globosum*	Eukaryote	XL1-Blue	pET28a	Heat (50 and 65 °C), salinity	Aggarwal et al. (2012)
DcHsp17.7	*Daucus carota*	Eukaryote	BL21 (DE3)	pET26b	Heat (46 °C), cold (16 °C)	Jung and Ahn (2022)
LimHsp16.45	*Lilium davidii*	Eukaryote	BL21 (DE3)	pET28b	Heat (45 °C) and cold (4 °C)	Mu et al. (2011)
TLHS1	*Nicotiana tabacum*	Eukaryote	MC1061	pBADNH	Heat (50 °C)	Joe et al. (2000)
OsHsp16.9	*Oryza sativa*	Eukaryote	XL1-Blue	pGEX-2T	Heat (47.5 °C)	Yeh et al. (1997)
OsHsp20	*Oryza sativa*	Eukaryote	BL21 (DE3) pLysS	pET32a	Heat (50 °C, 65 °C), salinity, dry, hormone	Guo et al. (2020)
RcHSP17.8	*Rosa chinensis*	Eukaryote	BL21 (DE3)	pET32a	Heat (50 °C), cold (4 °C)	Jiang et al. (2009)
TjHsp20	*Tigriopus japonicus*	Eukaryote	BL21 (DE3) pLysS	pCR T7 TopoN	Heat (54 °C)	Seo et al. (2006)

Ezemaduka et al. (2014) demonstrated that the heterologous expression of small HSPs derived from the nematode *Caenorhabditis elegans* allowed *E. coli* to grow at 50 °C, which is 3 °C higher than its maximum growth temperature. This finding is notable, particularly because *C. elegans* is a mesophilic organism (growth temperature range 15–25 °C), which is incapable of growing at such high temperatures (Gupta et al. 2007). This finding encouraged us to investigate the effect of introducing HSPs from organisms with high thermal resistance. Thermophilic and thermotolerant archaea also harbor small HSPs involved in cell maintenance at high temperatures (Laksanalamai et al. 2004; Lemmens et al. 2018; Roy et al. 2022). In addition, proteins from thermophiles and hyperthermophiles exhibit excellent tolerance not only to high temperatures, but also to other stresses, such as those caused by organic solvents and detergents (Owusu and Cowan 1989; Atomi 2005). In this study, we aimed to introduce 18 small HSP20s from 12 thermophilic and thermotolerant bacteria into *E. coli* (Table 2) and evaluate their effects on various physical and chemical cellular stresses.

**Table 2:** HSP20s used in this study

Name	Locus tag	Locus	Gene source	Length (aa)	Predicted MW (kDa)	Growth temperature of source (°C)			Reference on growth temperature
						Minimum	Optimum	Maximum	
G1	GK2146	2193409..2193852, BA000043	*Geobacillus kaustophilus* NBRC 102445	147	17.5	37	60–65	72	Sato et al. (2020)
G2	GK2159	2207139..2207603, BA000043		154	18.3				
G3	GK0256	Comp281982..282431, BA000043		149	17.3				
R1	Rmar_0989	1124320..1124766, CP001807	*Rhodothermus marinus* JCM 9785	148	17.1	54	65	77	Sato et al. (2020)
R2	Rmar_1296	1520995..1521426, CP001807		143	16.2				
R3	Rmar_1824	2132987..2133427, CP001807		146	17.0				
O1	Ocepr_0249	241959..242381, CP002361	*Oceanithermus profundus* DSM 14977	140	15.9	40	60	68	Sato et al. (2020)
O2	Ocepr_0921	936346..936759, CP002361		137	15.4				
O3	Ocepr_2086	2127398..2127871, CP002361		154	17.7				
TK	TKV_c23110	2214732..2215169, CP009170	*Thermoanaerobacter kivui* ATCC 33488	145	17.1	50	66	72	Sato et al. (2020)
KO	Kole_0885	933490..933942, CP001634	*Kosmotoga olearia* NBRC 109654	150	17.5	20	65	80	Sato et al. (2020)
TA	Theam_1062	1039880..1040386, NC_014926	*Thermovibrio ammonificans* HB1	168	19.6	69	75	80	Sato et al. (2020)
TE	tlr0873	809981..810418, NZ_CP032152	*Thermosynechococcus elongatus* BP-1	145	16.7	Not analyzed	57	Not analyzed	Onai et al. (2004)
TS	Tsedi_RS02180	49514..49939, NZ_VJND01000002	*Tepidimonas sediminis* NBRC 112410	141	15.8	45	45–50	60	Sato et al. (2020)
TM	TM0374	394075..394518, AE000512	*Thermotoga maritima* MSB8	147	17.6	55	80	90	Sato et al. (2020)
PH	CWI69_RS04835	1019265..1019720, NZ_PIPW01000001	*Pseudidiomarina halophila* NBRC 109833	151	16.8	4	30	55	Sato et al. (2020)
HT	HTH_0332	Comp336373..336804, AP011112	*Hydrogenobacter thermophilus* TK-56	143	17.1	50	70–75	77.5	Sato et al. (2020)
DT	Dester_0588	595231..595737, CP002543	*Desulfurobacterium thermolithotrophum* BSA	168	19.7	40	70	75	Sato et al. (2020)
CE	CELE_F52E1.7	Chromosome V:complement(8384799..8385929)	*Caenorhabditis elegans* (nematode)	148	17.6	16	20	25	Hedgecck and Russel (1975)
EA	ibpA	Comp3867009..3867422, CP009273	*Escherichia coli*	138	15.8	7.5	37	49	Ferrer et al. (2003)

## Materials and methods

### Strains and culture condition

The strains and plasmids used in this study are summarized in Table 3. *E. coli* strain One Shot TOP10 (Invitrogen, Carlsbad, CA, USA) was used for gene cloning, and Rosetta 2 (DE3) pLysS (Novagen, Merck, Darmstadt, Germany) or BW25113 was used for the expression of genes encoding each HSP20. *E. coli* strains or their transformants were cultivated in Luria Bertani (LB) medium at 37 °C at 180 rpm.

**Table 3:** Strains and plasmids used in this study

Name	Description	Experiment
Strains		
*Escherichia coli* TOP10	F^−^ *mcr*A Δ(*mrr*-*hsd*RMS-*mcr*BC) Φ80lacZΔM15 Δ *lac*X74 *rec*A1 *ara*D139 Δ(*araleu*)7697 *gal*U *gal*K *rps*L (StrR) *end*A1 *nup*G	For cloning
*Escherichia coli* Rosseta 2 (DE3) pLysS	F^−^ *omp*T *hsd*SB (rB- mB-) *gal dcm* (DE3) pLysSRARE2 (Cam^R^)	For expression
*Escherichia coli* BW25113	F^−^ DE (*ara*D-*ara*B) 567 *lac*Z4787(del)::*rrn*B-3 LAM^−^ *rph*-1 DE (*rha*D-*rha*B) 568 *hsd*R514	For expression
Plasmids		
pET28a	Empty vector (resistance, kanamycin; inducer, isopropyl β-D-thiogalactopyranoside)	For survival assay
pET28a-*ivy*	For expression of Ivy family C-type lysozyme inhibitor derived from *E. Coli* BW25113 (158 aa) which is a protein with similar molecular weight of HSP20	
pET28a-G1	For expression of *hsp20* from* Geobacillus kaustophilus*	
pET28a-G2	For expression of *hsp20* from* Geobacillus kaustophilus*	
pET28a-G3	For expression of *hsp20* from* Geobacillus kaustophilus*	
pET28a-R1	For expression of *hsp20* from* Rhodothermus marinus*	
pET28a-R2	For expression of *hsp20* from* Rhodothermus marinus*	
pET28a-R3	For expression of *hsp20* from* Rhodothermus marinus*	
pET28a-O1	For expression of *hsp20* from* Oceanithermus profundus*	
pET28a-O2	For expression of *hsp20* from* Oceanithermus profundus*	
pET28a-O3	For expression of *hsp20* from* Oceanithermus profundus*	
pET28a-TK	For expression of *hsp20* from* Thermoanaerobacter kivui*	
pET28a-KO	For expression of *hsp20* from* Kosmotoga olearia*	
pET28a-TA	For expression of *hsp20* from* Thermovibrio ammonificans*	
pET28a-TE	For expression of *hsp20* from* Thermosynechococcus elongatus*	
pET28a-TS	For expression of *hsp20* from* Tepidimonas sediminis*	
pET28a-TM	For expression of *hsp20* from* Thermotoga maritima*	
pET28a-PH	For expression of *hsp20* from* Pseudidiomarina halophila*	
pET28a-HT	For expression of *hsp20* from* Hydrogenobacter thermophilus*	
pET28a-DT	For expression of *hsp20* from* Desulfurobacterium thermolithotrophum*	
pET28a-CE	For expression of *hsp20* from *Caenorhabditis elegans* (nematode)	
pET28a-EA	For expression of *hsp20* from* Escherichia coli*	
pET28a-EB	For expression of *hsp20* from* Escherichia coli*	
pBAD30	Empty vector (resistance, ampicillin; inducer, arabinose)	For long-term heat assay
pBAD30-O2	For expression of *hsp20* from* Oceanithermus profundus*	
pBAD30-TS	For expression of *hsp20* from* Tepidimonas sediminis*	
pBAD30-PH	For expression of *hsp20* from* Pseudidiomarina halophila*	
pBAD30-CE	For expression of *hsp20* from *Caenorhabditis elegans* (nematode)	

### Construction of expression plasmids for small HSPs

Two types of plasmids were constructed for the expression analyses of HSP20s using the pET28a and pBAD30 vectors as the backbone. Primers used in this study are listed (Online Resource 1). Genomic DNA was extracted from cultured bacteria using a Wizard Genomic DNA Purification Kit (Promega, Madison, WI, USA). Genes encoding HSP20s were amplified using PrimeSTAR GXL DNA polymerase (Takara Bio, Osaka, Japan). For constructing pET28a-based plasmids, the amplicons and pET28a were digested with *Nco*I-HF/*Sac*I-HF or *Nco*I-HF/*Hin*dIII-HF. The GGA codon for glycine was added behind the start codon to avoid a frameshift in *Nco*I-HF site. The digested products were purified using the Wizard SV Gel and PCR Clean-up System (Promega) and ligated using T4 DNA ligase (Nippon Gene, Tokyo, Japan), following the manufacturer’s instructions. For constructing pBAD30-based plasmids, the amplified fragments were assembled using the NEBuilder HiFi DNA Assembly Master Mix (New England Biolabs, MA, USA). The constructed plasmids were introduced into *E. coli* TOP10 cells through a brief heat shock (42 °C, 45 s), and the transformants were cultivated for 14–18 h at 37 °C with appropriate antibiotics (50 µg mL^–1^ of kanamycin for pET28a-based plasmids; 100 µg mL^–1^ of ampicillin for pBAD30-based plasmids). Plasmid extraction from the transformants was performed using the Wizard Plus SV Minipreps DNA Purification System (Promega). The nucleotide sequences of the constructed plasmids were confirmed using Sanger sequencing.

### Verification of HSP20 expression in *E. coli*

The expression of HSP20s was confirmed using sodium dodecyl sulfate–polyacrylamide gel electrophoresis (SDS-PAGE). The pET28a-based plasmids were introduced into *E. coli* Rosetta2 (DE3) pLysS competent cells. The transformants were pre-cultivated overnight in LB broth including 50 µg mL^–1^ kanamycin and 17 µg mL^–1^ chloramphenicol. The culture was inoculated in fresh medium with the same antibiotics at 37 °C at 180 rpm. Isopropyl-β-D-thiogalactopyranoside (IPTG) was added at a final concentration of 0.2 mM in the early logarithmic phase (OD_600_ = 0.2–0.4) to induce the expression of each HSP20. After induction for 5 h, cells were washed twice with 50 mM Tris–HCl (pH 7.5) and resuspended in the same buffer (200 mg wet cells ml^−1^). Each suspension was subjected to cell disruption via sonication using an ultrasonic disruptor (UD-201; Tomy Seiko, Tokyo, Japan). The sonication conditions were 10 flashes (output 3, duty 75) and cooling for 10 cycles (30 s on ice). The crude lysate was centrifuged at 15,000 × *g* at 4 °C for 15 min to separate the soluble and insoluble fractions. The soluble fraction was heat treated at 70 or 80 °C for 30 min to evaluate the heat resistance of small HSPs briefly. The 5 µL of each fraction was subjected to SDS-PAGE, and proteins were visualized by staining the gels with Coomassie Brilliant Blue R250.

### Survival assay under abiotic stresses

The viability of *E. coli* cells was evaluated under extreme conditions (heat, cold, acidic, alkaline, and osmophilic). The transformants of *E. coli* strain Rosetta2 (DE3) pLysS were cultivated to the logarithmic stage (OD_600_ = ca. 0.7) at 37 °C in LB medium with 50 µg mL^–1^ kanamycin and 17 µg mL^–1^ chloramphenicol. The preculture (50 µL) was transferred into 5 mL of fresh medium supplemented with 0.2 mM IPTG, 50 µg mL^–1^ kanamycin and 17 µg mL^–1^ chloramphenicol. After induction for 15 h, 1 mL of the culture was centrifuged at 10,000 × g for 3 min. Cell pellets were washed twice with an equal volume of 0.8% sodium chloride (NaCl) and subjected to various stress conditions.

For heat and cold treatments, the washed pellets were resuspended in 1 mL LB medium (room temperature, pH 7, and 1% (w/v) of NaCl). The cell resuspension was transferred to 1.5 mL tubes, and the tubes were incubated at 52 °C (heat stress) for 30 min in a water bath or – 25 °C (cold stress) for 6 h in a freezer. Heat- or cold-treated samples were collected and serially diluted in 0.8% NaCl solution. To calculate accurate viability with or without the expression of *hsp20* genes, each diluted sample was spotted onto a solid LB medium without antibiotics or inducers. The colony-forming units (CFU) in each sample were counted in quintuplicate as biological replicates. Cell viability was calculated by comparing the CFU before and after treatment. As a negative control, *E. coli* cells transformed with empty pET28a (+) vector (Novagen) or pET28a-*ivy* coding Ivy family C-type lysozyme inhibitor in *E. coli* (158 aa), a protein with similar molecular weight of HSP20, were used. As a positive control, *E. coli* cells harboring pET28a with genes coding small HSPs from *E. coli* (ibpA or ibpB), containing functional domains similar to those of the HSP20s from thermotolerant bacteria, or small HSP from *C. elegans* (CE) were used (Table 2).

For acidic, alkaline, and osmophilic conditions, the washed pellets were resuspended in the following three different types of modified LB media: medium adjusted to pH 3 with HCl, medium adjusted to pH 11 with NaOH, or medium containing 10% (w/v) NaCl. Cell viability was evaluated in the same manner as that for heat and cold treatments.

Statistical analyses were conducted using the one-way ANOVA test to compare the control strain harboring pET28a-*ivy* with the HSP20-expressed strains, and *p*-values below 0.05 and 0.01 were set as significance thresholds for statistical significance in this study.

### Effect of long-term heat stress on cell viability

Viability of the recombinant *E. coli* expressing thermophilic HSP20 was tested after long-term heat treatment. Two *hsp20* genes (O2 and TS in Table 2) that enhance the thermotolerance of *E. coli* were evaluated. To avoid cell toxicity due to excess HSP20 production by the IPTG-induced systems, plasmids were reconstructed using pBAD30 containing an arabinose-induced promoter. A plasmid harboring the HSP17 gene from *C. elegans* and an empty vector (pBAD30) was used as positive and negative controls, respectively. Each plasmid was introduced into *E. coli* strain BW25113. The transformants were cultivated in LB medium containing 0.02% arabinose and 100 µg mL^−1^ of ampicillin at 37 °C overnight. The culture was exposed to high temperatures (52 °C) in a water bath for 5 days. Sampling was performed at 0, 0.5, 8, 24, 48, and 120 h to check cell viability using CFU on LB agar plates. To determine the proliferation ability of long-term heat-treated cells, the culture of each strain after 120 h was inoculated and cultured in LB medium at 37 °C and 180 rpm.

### Phylogenetic analysis of small HSPs

Phylogenetic analyses were performed using the amino acid sequences of small HSPs used in this and previous studies. In this study, 18 small HSP20s were selected from 13 thermophilic bacteria belonging to various taxonomic groups. The amino acid sequences were obtained from the Kyoto Encyclopedia of Genes and Genomes. Multiple sequence alignments were performed using Clustal Omega (https://www.ebi.ac.uk/Tools/msa/clustalo/). A phylogenetic tree was constructed by the neighbor-joining method using Genome Workbench version 3.5.0 and was visualized by iTOL version 6 (https://itol.embl.de/).

## Results and discussion

### Overexpression of HSPs

Eighteen HSP20s derived from 12 genera of thermotolerant bacteria were tested to evaluate their effects on the viability of *E. coli* under harsh conditions (Table 2 and Online Resource 2). We selected the bacterial genes from thermophiles and mesophiles belonging to the diverse taxonomic groups as follows: phylum *Pseudomonadota* (taxonomic group same as *E. coli*) [*Tepidimonas* (TS) and *Pseudidiomarina* (PH)], phylum *Thermotogota* (group containing mesophiles, thermophiles, and hyperthermophiles) [*Thermotoga* (TM) and *Kosmotoga* (KO)], phylum *Aquificota* containing chemoautotrophic thermophiles [*Thermovibrio* (TA), *Desulfurobacterium* (DT), and *Hydrogenobacter* (HT)], phylum *Deinococcota* [*Oceanithermus* (O1-3), halophilic thermophiles], phylum *Rhodothermota* [*Rhodothermus* (R1-3), halophilic thermophiles], phylum *Cyanobacteriota* [*Thermosynechococcus* (TE), photosynthetic thermophiles], and phylum *Bacillota* [*Geobacillus* (G1–3), spore-forming thermophiles]. Note that *Pseudidiomarina* (PH) is an exceptionally mesophilic genus.

**Fig. 1:**
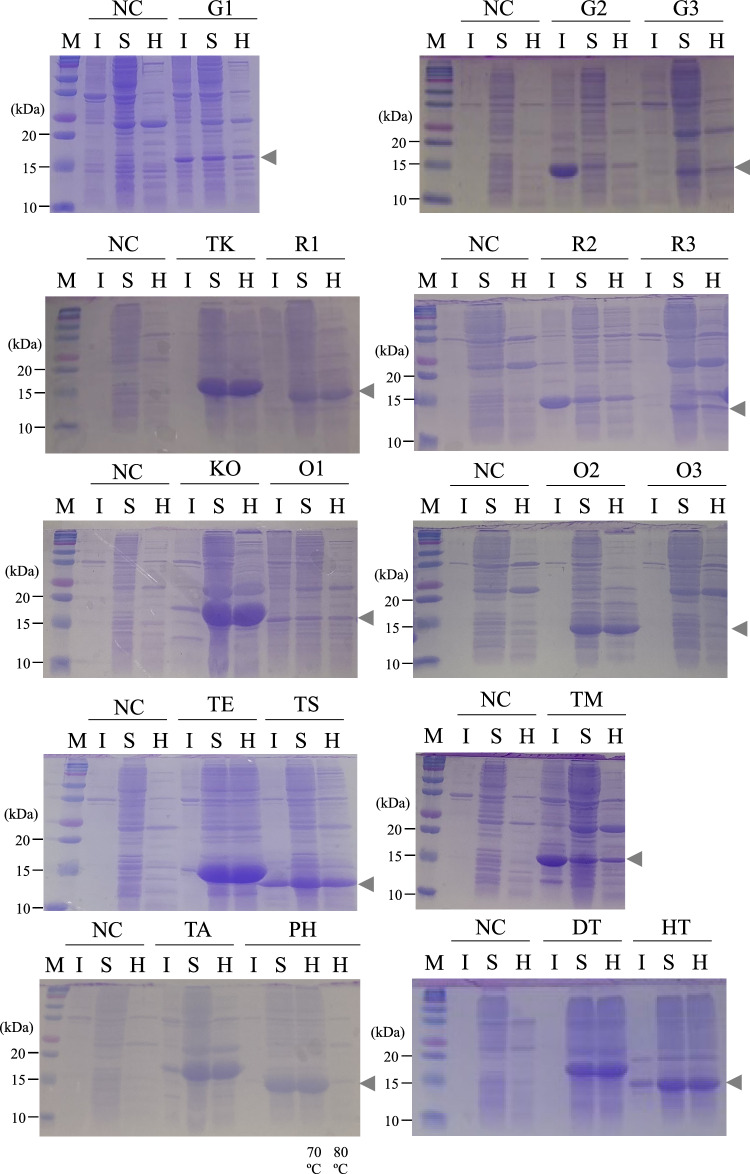
Sodium dodecyl sulfate–polyacrylamide gel electrophoresis analysis of 18 heat shock protein (HSP)20s from insoluble and soluble fractions expressed in *Escherichia coli*. Lane M, molecular mass marker (Precision Plus Protein Dual Color Standards, BIORAD); Lane I, insoluble fraction; Lane S, soluble fraction; Lane H, soluble fraction after heat treatment at 80 °C for 30 min. Only for *Pseudidiomarina halophila*, lane H indicates soluble fraction after heat treatment at 70 or 80 °C for 30 min, respectively

SDS-PAGE confirmed the presence of 17 HSP20s, except for O3 (OP2086 from *Oceanithermus profundus*), in the soluble fraction, suggesting their successful soluble expression (Fig. 1). This result is consistent with those of previous studies where various small HSPs from eukaryotes and prokaryotes were successfully expressed in *E. coli* (Table 1). In addition, the expression of each HSP20 did not seem to defect the growth of the host strains under the non-stress condition at 37 °C in comparison with that of control strains expressing a non-HSP20 protein with similar molecular weight (ivy; see Supplementary Method and Online Resource 3).

HSP20s derived from the thermophilic bacteria used in this study remained in soluble forms even after heat treatment (80 °C, 30 min), indicating their thermostable structure. In contrast, the HSP20 from mesophilic bacterium *Pseudidiomarina halophila* was found in the supernatant after exposure to 70 °C but not to 80 °C for 30 min. *P. halophila* is mesophilic, and its optimum growth temperature was the lowest among the bacteria tested in this study (Table 2). Therefore, the structures of the protein chaperones from thermophiles were more stable than those from mesophilic *P. halophila*, as expected.

### Temperature resistance of HSP20-expressed strains

**Fig. 2:**
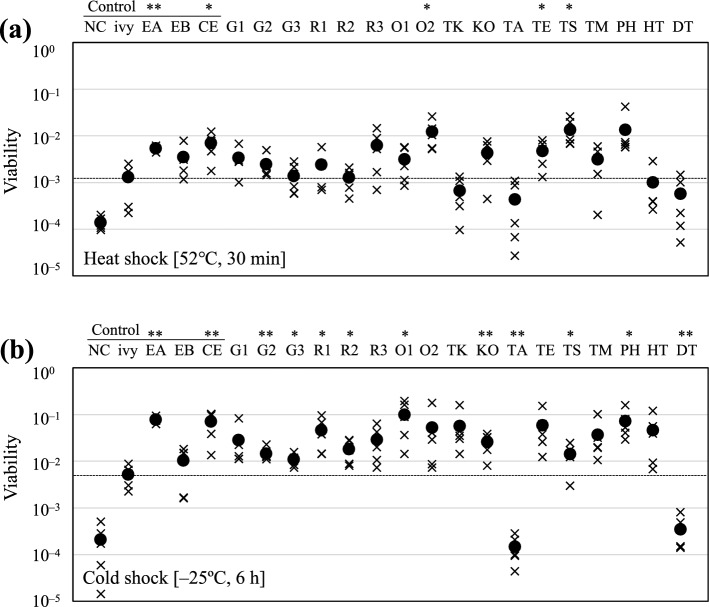
Viability of each mutant after temperature variation: **a** viability of each mutant after heat treatment (52 °C for 30 min). **b** Viability of each mutant after freeze–thaw treatment (− 25 °C for 6 h). The abbreviation of HSP20 is corresponding to that in Table 2. “NC” and “ivy” represent specific strains of *E. coli* Rosetta 2 (DE3) pLysS carrying different plasmids (pET28a and pET28a-*ivy*, respectively). The x-marks and filled circles represent the actual data points and the average values, respectively. Symbol mark indicates statistical differences with the control strain harboring pET28a-ivy by the one-way ANOVA method (*p-value < 0.05; **p-value < 0.01)

To reveal the effect of each HSP20 on elevated and cold temperatures, we determined cell viability after exposure to elevated temperature (52 °C, 30 min) and cold temperature (– 25 °C, 6 h). Most HSP20s from thermotolerant bacteria used in this study improved the resistance of *E. coli* to high and low temperatures as well as host’s small HSPs (EA and EB) (Fig. 2). Majority of the transformants demonstrated higher viability after heat treatment than that of the control strains harboring pET28a or pET28a-*ivy*. In particular, the expression of O2, TE, or TS increased the cell viability equal to or greater than that of EA, EB, and CE from mesophilic organisms, which allowed the growth of *E. coli* at temperatures higher than its maximum growth temperature (Ezemaduka et al. 2014). On the other hand, eight HSP20s improved the cell viability after cold treatment. The cell viability of the control strain with empty vector (NC) was significantly low, possibly due to the gradual freezing process from room temperature to – 25 °C. The control strain (ivy) overexpresses the small non-HSP protein also showed improved viability, suggesting that an excess of low-molecular-weight proteins in the cells may reduce cell stress like a compatible solute. These results are consistent with those of previous studies suggesting that HSP20s confer resistance to host cells at cold and high temperatures (Table 1).

However, TA and DT did not significantly improve stress resistance. Their amino acid sequences were 86.7% identical, which was higher than that (45.1% or less) between TA or DT and the other HSP20s used in this study (Online Resource 4). Alignment analyses suggested the amino acid residues, which are possibly involved in the chaperone activity of the proteins, in the α-crystallin domain of TA and DT (Online Resource 5). In addition, the molecular weights of TA and DT (approximately 20 kDa) were considerably higher than those of the other HSP20s (15–18 kDa). Both HSP20s were derived from thermophilic bacteria belonging to a similar taxonomic group (*Aquificae*). Therefore, these HSPs may be functionally different from other HSP20s.

### Multiple resistance of HSP20-expressed strains: acidic, alkalic, and osmophilic

**Fig. 3:**
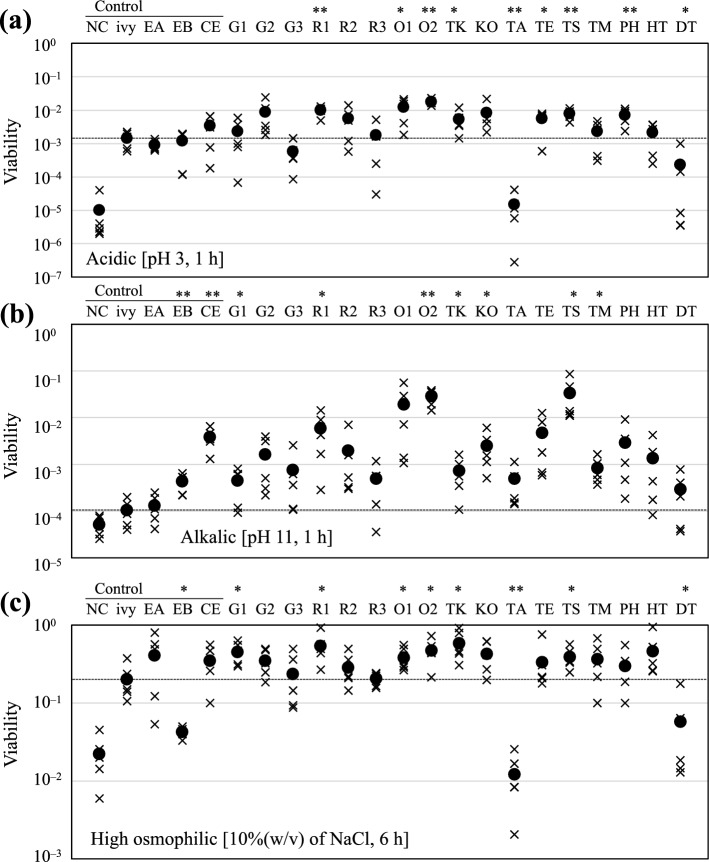
Viability of each mutant under multiple stress conditions: **a** viability of each mutant exposed to acidic condition (pH 3 for 1 h); **b** viability of each mutant exposed to alkaline condition (pH 11 for 1 h); **c** viability of each mutant exposed to high osmotic condition [10%(w/v) of NaCl for 6 h]. The abbreviation of each HSP20 is corresponding to that in Table 2. “NC” and “ivy” represent specific strains of *E. coli* Rosetta 2 (DE3) pLysS carrying different plasmids (pET28a and pET28a-*ivy*, respectively). The faction marks and filled circles represent the points of each measurement and the average values, respectively. Symbol mark indicates statistical differences with the control strain harboring pET28a-ivy by the one-way ANOVA method (*p-value < 0.05; **p-value < 0.01)

To reveal the effect of each HSP20 on the other stresses except for temperature, we investigated the cell viability after exposure to acidic (pH 3, 1 h), alkalic (pH 11, 1 h), and hyperosmotic (10% NaCl, 6 h) conditions (Fig. 3). Compared to the control strains harboring pET28a (NC) or pET28a-*ivy* (ivy), HSP20 also improved the viability of *E. coli* to multiple stresses other than extreme temperatures. For seven HSP20s (R1, O1, O2, TK, TE, TS, and PH), cell viability under acidic conditions significantly increased than that of the control strain with pET28a-*ivy* and were more than 100-fold higher compared to that of the control strains harboring empty vector (NC), suggesting that most HSP20s including small HSPs from *E. coli* enhanced the acid tolerance of *E. coli* (Fig. 3a). Several HSP20s also improved the cell viability under alkaline conditions (Fig. 3b). Especially, O2 and TS improved cell viability with statistical significance by more than 100-fold in comparison with the ive-expressing strain. In addition, some HSP20s, including O2 and TS, enhanced the viability of *E. coli* after exposure to high osmotic pressure (10% [w/v] NaCl) (Fig. 3c). Two types of HSP20s, O2 and TS, successfully improved tolerance to a variety of stresses in *E. coli*. On the other hand, TA and DT did not improve the viability under most stress conditions in comparison to the other HSP20s. Although we have identified the two amino acid residues (alanine in positions 149 and 150) conserved specifically in TS and O2 and five residues (Positions 103,106, 122,128, and 156) found only in TA and DT (Online Resource 5), the impact of these residues on stress tolerance remains unclear.

### Cell viability under long-term heat stress conditions

**Fig. 4:**
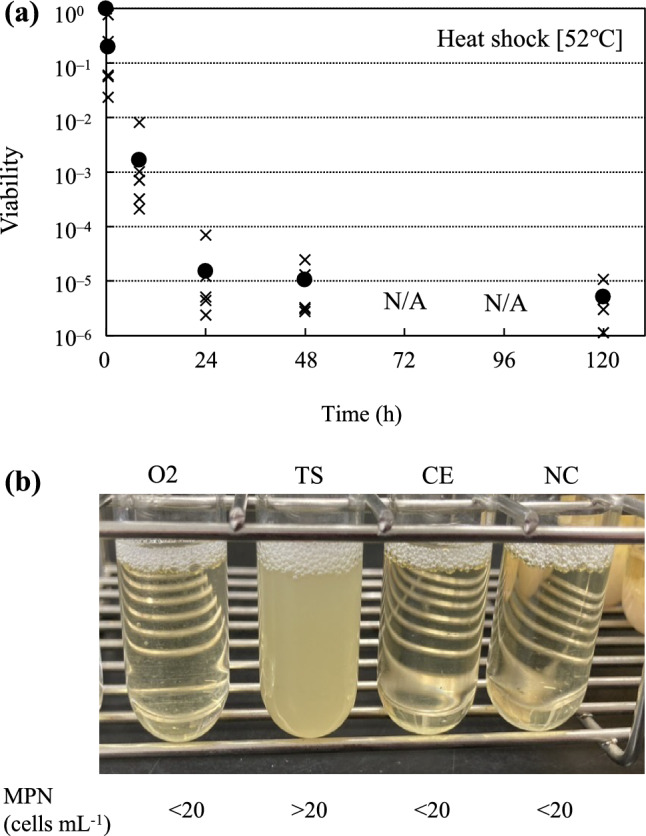
Cell viability after long-term heat treatment. **a** The time course of cell viability of strain BW25113 harboring pBAD30-TS after long-term heat treatment (52 °C). The faction marks and filled circles represent the points of each measurement and the average values, respectively. Most probable number (MPN) shows the estimated number of viable cells in long-term heat-treated samples. **b** The cultivation results at 37 °C for 3 days using strains exposed to 52 °C for 5 days. N/A indicates the time point of no measurement

We verified whether the maximum growth temperature of *E. coli* could be increased by HSP20s by O2 or TS expression. Although two sets of expression systems, pET28a/Rosetta 2 (DE3) pLysS and pBAD30/BW25113, were tested, HSP20 expression did not affect the maximum growth temperature (47 °C) of *E. coli* (Online Resource 6). The maximum growth temperature was consistent with the value in the previous study (Schink et al. 2022). In contrast, for strain BW25113 harboring pBAD30-TS, some viable cells were identified after long-term heat treatment (52 °C, 5 days) using the colony-forming assay (Fig. 4a). In addition, the strain could proliferate at 37 °C after the treatment for 5 days (Fig. 4b), although the other strains, including negative (with empty vector) and positive (expressed CE) control strains, did not proliferate. Therefore, HSP20 from *Tepidimonas* affords *E. coli* to survive after prolonged (> 100 h) high-temperature stress.

We further investigated how HSP20 (TS) contributes to the homeostasis of *E. coli* under severe conditions. Compared with the other thermophiles used in this study, TS (beta-proteobacteria) is phylogenetically similar to *E. coli* (gammaproteobacteria). Therefore, the effective protection of *E. coli* cellular proteins by HSP20 (TS) may be due to their phylogenetic proximity and compatibility with structurally similar proteins. We intend to elucidate the detailed mechanisms of this phenomenon in future studies.

In conclusion, we demonstrated the improvement in *E. coli* stress tolerance by the heterologous expression of HSP20s from thermotolerant microorganisms. Expression of several HSP20s enhanced stress tolerance in *E. coli* as much as or more than those of ibpA and ibpB from *E. coli*. In particular, *E. coli* with thermotolerant HSPs, such as O2 and TS, exhibited remarkable stress tolerance, comparable to that of *C. elegans* HSP20. These findings indicate the potential of thermotolerant HSPs as molecular tools for improving stress tolerance in *E. coli*.

### Supplementary Information

Below is the link to the electronic supplementary material.

Supplementary file1 (PDF 833 KB)

## Data Availability

All experimental data are available upon request.

## Abbreviations

CFU: Colony-forming units
HSP: Heat shock proteins
IPTG: Isopropyl-β-D-thiogalactopyranoside
LB: Luria Bertani
SDS-PAGE: Sodium dodecyl sulfate–polyacrylamide gel electrophoresis
