# Supplementary material for: Effects of small heat shock proteins from thermotolerant bacteria on the stress resistance of Escherichia coli to temperature, pH, and hyperosmolarity
Source: Extremophiles. 2024 Jan 22;28(1):12. doi: 10.1007/s00792-023-01326-y (PMC10803503; doi:10.1007/s00792-023-01326-y)
Supplement: Supplementary file 1 — Supplementary file1 (PDF 833 KB) [file 792_2023_1326_MOESM1_ESM.pdf]

Extremophiles

Online Resource

Effects of small heat shock proteins from thermotolerant bacteria on the stress resistance of *Escherichia coli* to temperature, pH, and hyperosmolarity

Correspondence author
Yu Sato
[1] Division of Agricultural Sciences, Graduate School of Sciences and Technology for Innovation, Yamaguchi University, Yamaguchi, Japan
[2] Graduate School of Science and Technology for Innovation, Yamaguchi University, Yamaguchi, Yamaguchi 753-8515, Japan
[3] Research Center for Thermotolerant Microbial Resources, Yamaguchi University, Yamaguchi 753-8515, Japan
E-mail: yusato@yamaguchi-u.ac.jp

Supplementary Methods

Growth profiles of HSP20-expressed strains under non-stress condition

Growth curves of *E. coli* expressing thermotolerant HSP20 were obtained under non-stress condition. The pET28a-based plasmids containing each *hsp20* gene, gene coding non-HSP (Ivy family C-type lysozyme inhibitor), or none (empty vector) were transformed to the strain Rosseta 2 (DE3) pLysS. Each transformants were cultivated in LB medium containing 0.2 µM IPTG, 50 µg mL^{-1} of kanamycin, and 17 µg mL^{-1} of chloramphenicol at 37 ºC for 14-16 h. The 50 µL of culture was transferred to new 5 mL of fresh LB medium containing same inducer and antibiotics. Cell growth was monitored using a rocking incubator equipped with a spectrophotometer (TVS062CA; Advantec Toyo Co Ltd., Tokyo, Japan). Culture condition was at 37 °C with a rocking shake at 50 rpm. The OD_{660} value was recorded every 30 min. Growth rate (µ) was calculated between the individual incubation periods (t_1 and t_2) with an assumption of exponential growth; *i.e.*, $\mu\ (h^{-1}) = (\ln Nt_2 - \ln Nt_1)/(t_2 - t_1)$, where Nt_1 and Nt_2 indicate cell density, respectively. Doubling time (DT) was calculated in exponential growth phase (OD_{660} = 0.2-0.9) using the following equation, DT (h) = log2 / µ.

Determination of the maximum growth temperature

Growth curves of *E. coli* BW25113 expressing thermophilic HSP20 were also obtained at high temperatures close to the maximum growth temperature. We tested growth of the strains harboring pBAD30 containing *hsp20* gene (O2 or TS in Table 2) that enhanced the thermotolerance of *E. coli* at 52ºC. A plasmid harboring the *hsp17* gene from *C. elegans* and an empty vector (pBAD30) were used as positive and negative controls, respectively. Each transformants were cultivated in LB medium containing 0.02 % arabinose and 100 µg mL^{-1} of ampicillin at 37 ºC for 14-16 h. The 50 µL of culture was transferred to new 5 mL of LB medium

containing same inducer and antibiotic. Culture condition was at 46, 47, and 48°C with a rocking shake at 50 rpm.

Cell growth was monitored using as shown in the previous paragraph.

Online Resource 1 Primers used in this study

Name	Sequence (5' to 3')
(Ligation)	
G1_NcoI_F	ATA**CCATGG***GA* GCGTTAATTCCGTATGACCCGT
G1_SacI_R	CAT**GAGCTC**TTAATGGAACTCGATATCAACGCGTTTTTCG
G2_NcoI_F	ATA**CCATGG***GA* AATGAATCGTTTCAACCACCAGCC
G2_SacI_R	CAT**GAGCTC**TCAATCGATATCGATATATTTCCGTTTTTGCGG
G3_NcoI_F	ATA**CCATGG***GA* ACCAACGAAAATCCATTTATGCCGTT
G3_SacI_R	CAT**GAGCTC**TTATTCGCTTTTTTTGATGACAATTTTCTTTTTCGG
R1_NcoI_F	ATA**CCATGG***GA* ACGAGCCTGGTTCGCTTTTC
R1_SacI_F	CAT**GAGCTC**TTAGCTGATCTCGATCCGACGC
R2_NcoI_F	ATA**CCATGG***GA* GCCGACCGCATTTATTTCC
R2_SacI_F	CAT**GAGCTC**TCACTCCACGGTGATCTTCCG
R3_NcoI_F	ATA**CCATGG***GA* GCCGAGGTGATGCGCTATAC
R3_SacI_F	CAT**GAGCTC**TTACGAGATCTTGATCCGCACCGG
O1_NcoI_F	ATA**CCATGG***GA* ATGCGTTGGGATCCGTTCAAG
O1_SacI_F	CAT**GAGCTC**TCAGGACTCGACCTCGACCTG
O2_NcoI_F	ATA**CCATGG***GA* GAACTGGAACGAAGCGACC
O2_SacI_F	CAT**GAGCTC**CTAGCTGGGTTCGATGGGCAC
O3_NcoI_F	ATA**CCATGG***GA* GCTCTGGTGAAGCGCGAGA
O3_SacI_F	CAT**GAGCTC**TTACTGAGCGACCGAGATGGGGATC
KV_NcoI_F	ATA**CCATGG***GA* AGCCTTATGAGACGAGG
KV_SacI_R	CAT**GAGCTC**TTATTCTATATCTATTTTTCTTCCCTTTGGT
KO_NcoI_F	ATA**CCATGG***GA* TTGGTTCCAAAGAAGAACGTTTC
KO_SacI_R	CAT**GAGCTC**TTACTCAACTTTCACACTTACCGTTTCT
TA_NcoI_F	ATA**CCATGG***GA* TTCGGAGGATTTTTCGGAAGGAGA
TA_SacI_R	CAT**GAGCTC**TTACTTCTCTTCCTTACCCTTACCCTTCTTG
TE_NcoI_F	ATA**CCATGG***GA* GCACTCGTTCGTTGGGAAC
TE_SacI_R	CAT**GAGCTC**TTAGGCAAGGCTCACTTTGACGAC
TS_NcoI_F	ATA**CCATGG***GA* AGCAACCTGATTCCGCGC
TS_SacI_R	CAT**GAGCTC**TCACTCGATGCGGATGCGCT
TM_NcoI_F	ATA**CCATGG***GA* CTTCTTGGAAGAAGAGAAGACATCTTC
TM_SacI_R	CAT**GAGCTC**TTACTCCTGAACCTCCACCTCGATC
PH_NcoI_F	ATA**CCATGG***GA* GCAACTATAGATTTATCACCACTTTACCGTA
PH_SacI_R	CAT**GAGCTC**TTACTTTGCAGAATCTTCCAGCAAGTTCAG]
HT_NcoI_F	ATA**CCATGG***GA* AGAAGGAGCATCGCGTTGTG
HT_SacI_R	CAT**GAGCTC**TCATTTGATCTCTATCTTTTTTTCCTTTGATACCTC
DT_NcoI_F	ATA**CCATGG***GA* TTTGGAGGATTCTTTGGAAGAAGAGG
DT_SacI_R	CAT**GAGCTC**TTATTTTTCTTCCTCATTTTTCTTATCTGTTTCATC
CE_NcoI_F	ATA**CCATGG***GA* GATCGTCGTTTTCCGCCTTTTAG
CE_HindIII_R	CAT**AAGCTT**TTAGTTGCGTTTCGGAACAATGGTAATCG
EA_NcoI_F	ATA**CCATGG***GA* CGTAACTTTGATTTATCCCCGCTTTACCGT
EA_HindIII_R	CAT**GAGCTC**TTAGTTGATTTCGATACGGCGCGGT
EB_NcoI_F	ATA**CCATGG***GA* CGTAACTTCGATTTATCCCCACTGATGC
EB_HindIII_R	CAT**GAGCTC**TTAGCTATTTAACGCGGGACGTTCG
IVY_NcoI_F	ATA**CCATGG***GA* GGCAGGATAAGCTCGGGAGGAATG
IVY_HindIII_R	CAT**GAGCTC**TTATTTAAAATTAAAGCCATCCGGATGGTTTTCCAG
(Gibson Assembly)	
pBAD18_F	GCTGTTTTGGCGGATGAGAGAAGATT
pBAD18_R	CAGTAGAGAGTTGCGATAAAAAGCGTCAG
CeHSP_18F	CGCTTTTTATCGCAACTCTCTACTGATGGATCGTCGTTTTCCGCCTTTTAG
CeHSP_18R	ATCTTCTCTCATCCGCCAAAACAGCTTAGTTGCGTTTCGGAACAATGGTAATCG
PhHSP_18F	CGCTTTTTATCGCAACTCTCTACTGATGGCAACTATAGATTTATCACCACTTTACCGTA
PhHSP_18R	ATCTTCTCTCATCCGCCAAAACAGCTTACTTTGCAGAATCTTCCAGCAAGTTCAG
Op2HSP_18F	CGCTTTTTATCGCAACTCTCTACTGATGGAACTGGAACGAAGCGACC
Op2HSP_18R	ATCTTCTCTCATCCGCCAAAACAGCCTAGCTGGGTTCGATGGGCAC
TsHSP_18F	CGCTTTTTATCGCAACTCTCTACTGATGAGCAACCTGATTCCGCGC
TsHSP_18R	ATCTTCTCTCATCCGCCAAAACAGCTCACTCGATGCGGATGCGCT

Bold and italic characters indicate the restriction sites and dinucleotide added to NcoI site, respectively. Double underlines indicate gibson assembly site in primers.

Online Resource 2 Nucleotide sequences of HSP20s used in this study

Name	Sequence (5'-3')
G1	atggcgttaattccgtatgacccgttccggcacttggaatcgattcgccgggatatgaaccgctttttttgctagtgattttccatcgctgttcactcatatggatgagcagcactggatgccgcgcatcgacatgcatgaaacggccaacgaatacgtcgtttcgtgcgatttaccggggctggagcggaaagaggatgtgcacattgacgtgcagaacaacatgttgaccatcagcggcacgattcaacgccaccacgatgtaagagaagaacaaatgcaccggcgcgaacgcttctttggtcgcttccagcgttcgatcaccctgccggcggatgcggcgacggaaaacattcgcgcgacgtacaaaaacggcgtgcttgacattcatatcccgaaaacaacgacaggaacgaaaaaacgcgttgatatcgagttccattaa
G2	atgaatgaatcgtttcaaccaccagccaatgggggaggccatcacccgttccatcatttgcggaaaatgatgaatcaatggtttgatgaacggccgctgcaaaaactgtttgaaacgctcgatgactatttcgcacaaacgtttgctgcagcgtacattccgatcgaggtgaaagagacgaagcacgattaccaaatcattgtccggctgcctgacatcaaaagggaacaaatcagcttgcaatggcaggaagatgggctgcagctgatcgtcgatcacaatgaaacaattgaatcggcagatcatcacggccatgtatacgaacgccgccacgcacggcggcgcgtggcgaggctgattccgtttccgtatccggtcgctgagcatgaggtgaaagcctcgtttcaaaatgggacgctcaccatccagctgccgcaaaaacggaaatatatcgatatcgattga
G3	atgaccaacgaaaatccatttatgccgttttccgattggacaaaacattggcaacaatttttccaaaatgatttttggggaagcatccagccgttgctgccgtcatcaaactcaaacaaaccatcatccggcatgaacatttataaaaaagacaacgagctgctcgttgtcatcagcctcccggggctcgaaaaaatcgaagatgcagaagtgtatgtatcatacaaaacgttggaagtgaaggcaaccattaatctaaacttcaaagggtttgagctagtagaagaaggccttttccaaggccaattccaaaaaacaatcccactgccgttcgccgtcaaagaggaccgcatcgaagcaacataccataacggcctgttgttcatccacctgcatcgacttatccctgacgagccgaaaaagaaaattgtcatcaaaaaaagcgaataa
R1	atgacgagcctggttcgcttttcgccgatgacggagctgcgtcggctgcagcgtgagatggaccgcctgttcgatacgttcttcgggcgtgaagtggaagcggccgaagagacggcgccggtgacctgggtgccgcgggccgacctggccgaaacggacgatgcctacctgattcagctcgacgtgcccggcatgaacaaggacgagctgtcggtgacctatcacgacggcaccctcacggtcagcggtgagcggaagtcggagaccaaggaagagaagccgaactacatccgggtcgagcggagctacggccgcttctatcggagcttcacgctcccgaaggcggtcgatgaaaagaacatcgaagccaagtacgagaatggcgtgctgacgatccgcgtgcccaaggccgaaggcagcaaggcgcgtcggatcgagatcagctaa
R2	atggccgaccgcatttatttccccggtttcacaaccctgcaacgcgagatgaaccggctgtttgacgaatttctgcggggtgctgaagcgacgaccgaagcgccggctacctggacgccccgcgcggatctgtcggagacggccgaagcctacctgatccggatggatctgccgggcgtggccaaagaaagcctggacatccagttcaacgaaggcgtactgaccgtctcgggtgaacgaccggccgagtacgaaggggatcaggaaacggtgcggcatgtggagcggccacacggccggttcttccggagcttcacgctgccgcagacgattgatccggccggcatcaaggccgaaatgcgcgaaggggtgctgaccatccgtattccgaagctggccgcccaccagccgcggaagatcaccgtggagtga
R3	atggccgaggtgatgcgctatacgtcgccgacgtcgctgctgtcggagctgcagcgggaagtggaccggctgttcgagaacttcttcggaggctggctgcggcctgaggtcgagtcggcggtctggacgcccacggtggacctgctggagacggacgacgcttacctcatctacatggacctgcccggcgtgaaccgggaccaggtcacgatcaccttcgagaacggcacgctgcaggtgagcggtgagcgggtgcagcccgagcacaaggacgcgcagtatcatcggatggagcggtggtatggccgcttcttccggtcgttcaacctgggccagaacgtgaatccggataagatcaaggcccacttcgagaacggcgtccttgtgatcgaggcgccgaagacggaggagagcaagccggtgcggatcaagatctcgtaa
O1	atgatgcgttgggatccgttcaaggaaatcgaggaactgcaggagcggctgggccgcggtctgggcatgggccggggggcgcgcggcgaacagaccctggcgcccctggtggacgtctgggaggacgagcacggactccacttcgcggtctacctgcccggcatcgaacccgacaaggtcgaggtcacggccgaggaggagaccctgacggtcaaggccgaacggcccttcgagaagcaggagaacgtggcctaccaccgggtcgaggggccgtacggcaccttcgtgcgcagcttctcgatccccgcggtctacgacctgggcaaggtggccgccaagttcaagaacggcgtgctctacctgaccgttccgcgcgcggaggagaccaagccgcgcaagatccaggtcgaggtcgagtcctga
O2	atggaactggaacgaagcgaccgctgggcggccatcgaaaagctgatcgaactcaagcggcgtatcgaggcgctggagtcgcgcttcggggcggacgccctcgccgactgggctccgcccgtcgacgttctcgacgagggcgaggcctaccgaatcctggtggacgtgcccggggtcaagccccgaggacctcgagctgcaggaagaggggcgcacgatcacgatcgccggcgtgcgtcacgaacccgaggcgcgctacgtcacccgcacccgcccgcacggttacttccgccgggcctttacgctgcccgagccgatcgtcgagggcgcggccgaggccagcctgaagcaggggggtgctcgagatccgcatccccaaggcgcaggggcgcgcggtgcccatcgaacccagctag
O3	atggctctggtgaagcgcgagattcgcccgaccgctgaactcacccccttccgcacctggggtccgatgagcttattcgacgagttcaaccggctcttcgaggagaccttgggcgacctcggccgcgtgaccgcgcccagcacctacgtggcgccggtggacctctacgagaccgacgacgcgctggtgctggagatggcggtccccgggctgaccgcggaggagatcgacatcagcctcgagggcaacaagctgacgatccgcggtgagcacaagccggtcgaggatcagggcgtgcgccgctactacctgcaggagatcccgcacggcaccttcgtccgcagcttcaccctgccggtggagatcagcagcgacgaggtgaaggccgaattcaagaacggcatgctcaagctgaccatgcccaaggtcgagaccgcgcgcgcgccaagcggatccccatctcggtcgctcagtaa
TK	atgagccttatgagacgaggacgcgactggtgggattggcccttcgatttcaatattagaaatttgccaagcatttttgatgtgaatttcccatctatttcaggtctattttctcggccaagagtggacatcaccgaatctgaaacagaaatagtggcaacagctgaactaccgggcgttgacaaaaaagacattgaaataaatgtctatgacaatatactggaaataaaagggcaaaccacagtagatgaagaaagagaagataaaaattattacatgagagaaagatattacggaagttttgccagaagaatagaattacccgcagaagtagaccctgaaagaactactgctaaatttgaaaacggcattctcaaaatcacaatgccaaaattacatccaagcaaaccaaagggaagaaaaatagatatagaataa
KO	atgttggttccaaagaagaacgtttctgaactcttcagaccctttgaggaaattcagaaagagatcgataagcttttcagcgaagcctttagaggactcgatgtaagaagaggagagtacggcatgctcatccccgaagtcgatatctacgaaacggacgatgccatatttgtggaaatggaagtacctggcataaagaagaaagatctcgaaatcaaaattgaagatggaatcctgacaatcaaaggagagaaatcctctgaaaaagatgacaaatccagaaactaccatctctacgagcgttcctatggtatgttccagagagctttcagattgcccgacagtatcgatacgaccaaagttaaagcaaaatacgaagacggcgtcctgaaaatcgaattgcctaagaaggaagaagtcaagaaagaaacggtaagtgtgaaagttgagtaa
TA	atgttcggaggatttttcggaaggagaaggggattcgggtttgacccctttgaggatatcttcaggcagatggagatgatgatggccgagatgatgcggggtttcggcagggagttccccggctttgagatgggatttgagccccgcatcgagatgtacgagacccccgatgaaattgttgtaagggccgagatgcccggtctcgataaggacagcatagacgttaaagtaaggggcaactacctgattataaggggcgttaagaagcaggaggagaaagaggagaaggacaacgttttcttcagcgagaccttctacggcgagttccagagggtaattcccctgccggttgaggttaaagaagacggcatagaggcttactacgataaaggaatccttgagattaggcttcccaaggccgatgccgcccgtaaagaggtgaagattatcgttaaagagcccgaggaggataagggcaagaagggtaagggtaaggaagagaagtaa
TE	atggcactcgttcgttgggaaccgttccgcgaaatcgatgcaattcaacgccaaatgaaccgtctattcgatgaattgattcccttgacggagcgacgcagtgatctgagttttctgcctgctgcagaactggaagaaacccctgaagcgcttcttttgaaagtagagcttccgggaatggatcccaaagacattgacgtccaagttacagcggaagccgtttccattagtggcgaacgcaagtctgaaaccaaaactgaaacagagggcatgaaacggacagaattccgctatggcaaattccaacgggtcattcctctgccagttcggattcaaaacaccagtgttaaagctgaatacaaagatggcatcctgcacctgaccctgccgaaagcggaagaagagaaaaaccgcgtcgtcaaagtgagccttgcctaa
TS	atgagcaacctgattccgcgccgcatgagcctgttcgatgagttcttccgtgacctggcgccgggcttctacattcgcccgctgcacggcgacccgctgccgcagcagatcaaggtcgaagtcaaggagaacgaccaggcctacgtcatctcggccgagatgcccggcgtcggcaaggacaacatccacgtcacggtggagaacaacgtcgtgacgatcgccgccgaggtcaagcaggaggacgccaagcgcgacggcgagaaggtgctgcacagcgagcgctactacggccaggtgtcgcgcagcttcgcgctgcccagcgacgtcgacgagtccgccgccagcgccaagtacgacgacggcgtgctgaccctgacgctgccgaagaaggtggcggtggccggcaagcgcatccgcatcgagtga
TM	atgcttcttggaagaagagaagacatcttcaggccgttcagggaactccagagggagatcgacaggctcttcgatgacttcttcaggacggaggtcagacccgctaaggagttctttgcaccagacatggatgtgtacgaaacagacgatgaagtagtgatagaagtagaaatcccaggcatagacagaaaagacgtcaaaataacggttgaagagaacattttgaagatctctggtgaaaagaaactcgaaagagaacagaaaggtaaaaactattactacgttgagagaagcgctggaaaattcgaaagggcaatcagactcccagactacgttgacgtagaaaagatcaaagcagagtacaagaacggtgttctcacgataagggtaccaaagaaagaagaaaggaagaagaaagtgatcgaggtggaggttcaggagtaa
PH	atggcaactatagatttatcaccactttaccgtagtagcattggatttgatcgcatggcacgtttgctggactccgcaatgcgcgcggacggcaacgcagcaggcggctatcctccctataatatcgaagttattggcgagaatcgctacgctattacgctggccgtagctggtttttccgatgacgaactcagcatcgaagtcgagaatggcatgctgaaaatccagggccgcaaggaagatgaaaacgaagaacggcaatacctgcacaaggggattggcttccgtagcttcgagcgcaaattcaacctggctgatcatgttgaagtgaaaggcgcgaaaatggaaaacggtctgttaaccgtaggccttgagaaggttattccggaggcgatgaaacctcggaaaatcgaaatcggcaaatcgctgaacttgctggaagattctgcaaagtaa
HT	atgagaaggagcatcgcgttgtggaaccccttttgctgaactggagagaattagaagagagtttgacaggcttattgaagaaatgtggccaagagaagaggtagaaagagcttttgcacctgctgttgagatgtacgaaacggataatgaaatagttgttaaggcagaattgcccggtgtgaaaaaggagaacatcgaggtgagtataaaggacaatactctacacataagaggagagaagaaggaagagcgcgaggaaaagactgaaaccattcatcgcttagagagagtttacggaaagtttgaaagggtgttgactctgccggtagatgtgaaggctgaagaggtgaaagctgagtataaggacggtattttggaaataaggctccccaagtctgaggtatcaaaggaaaaaaagatagagatcaaatga
DT	atgtttggaggattctttggaagaagaggaggatatgatccttttgaagatatcttcagacagttccaggaaatggaaagatttatgtctgaaatgatgagaggttttggaagagaatttcctggatttgaagcaggttttgagccacgaatagaaatgtacgaaactccagatgaggtagttgtaaaggctgaaatgccaggtcttgacaaagacagtatagacgttaaagtaagaggaaactacctcattataaaaggtgttaaaaagcaagaaaagaaagatgagaaggagaacgttttctttagtgaaagcttttacggagagttccaaagggtaatacctttaccagtagaagtaaaagaggaaggaatagaagcaatttatgataagggaatacttgaaattagacttcctaaagcagattctgcaaggaaagaagttaagattattgttaaagaacctgaaaaagatgaaacagataagaaaaatgaggaagaaaaataa
CE	atggatcgtcgttttccgccttttagcccgtttttcaatcatggtcgtttcttcgatgatgtggattttgatcgtcacatgattcgtccgtattgggcagatcagaccatgctgacaggtcatcgtgttggtgatgcaattgatgtggtgaataacgatcaagagtataacgttagcgttgatgtgagccagtttgaaccggaagaactgaaagttaacattgtggataaccagctgatcatcgagggtaaacataatgagaaaaccgataaatatggtcaggtggaacgtcattttgtgcgcaaatataacctgccgaccggtgttcgtcctgagcagattaaaagcgaactgagcaataatggtgttctgaccgtgaaatacgagaaaaatcaagaacagcagccgaaaagcattccgattaccattgttccgaaacgcaactaa
EA	atgcgtaactttgatttatccccgctttaccgttctgctattggatttgaccgtttgtttaaccacttagaaaacaaccagagccagagtaatggcggctaccctccgtataacgttgaactggtagacgaaaaccattaccgcattgctatcgctgtggctggttttgctgagagcgaactggaaattaccgcccaggataatctgctggtggtgaaaggtgctcacgccgacgaacaaaaagagcgcacctatctgtaccagggcatcgctgaacgcaactttgaacgcaaattccagttagctgagaacattcatgttcgtggtgctaacctggtaaatggtttgctgtatatcgatctcgaacgcgtgattccggaagcgaaaaaaccgcgccgtatcgaaatcaactaa
EB	atgcgtaacttcgatttatccccactgatgcgtcaatggatcggttttgacaaactggccaacgcactgcaaaacgccggtgaaagccagagcttcccgccgtacaacattgagaaaagcgacgataaccactaccgcattacccttgcgctggcaggtttccgtcaggaagatttagagattcaactggaaggtacgcgcctgagcgtaaaaggcacgccggagcagccaaaagaagagaaaaaatggctgcatcaagggcttatgaatcagccatttagcctgagctttacgctggctgaaaatatggaagtctctggcgcaaccttcgtaaacggtttactgcatattgatttaattcgtaatgagcctgaacccatcgcagcgcagcgtatcgctatcagcgaacgtcccgcgttaaatagctaa

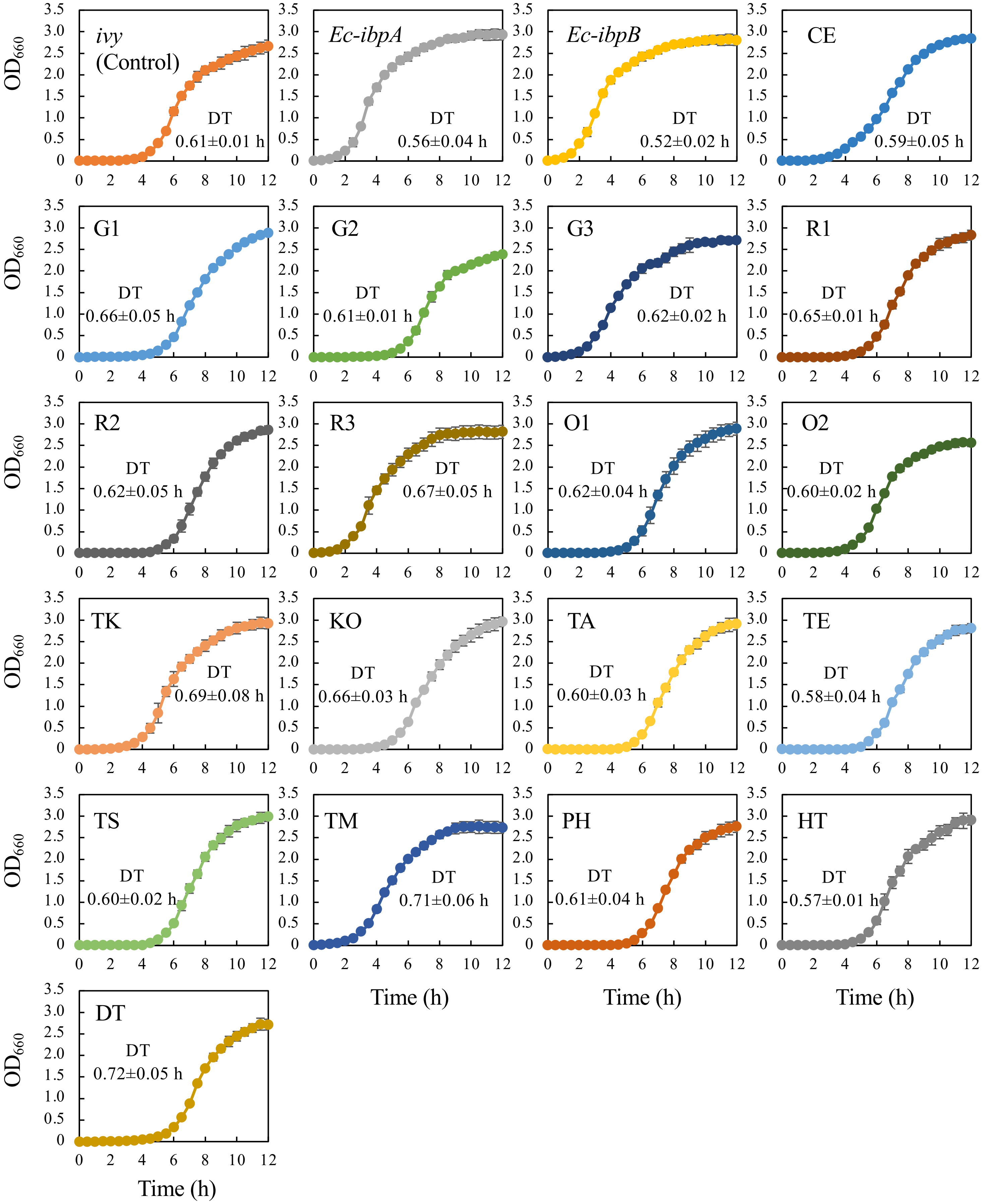

Online Resource 3 Growth curve of each strain harboring pET28a-based plasmids in absence of stress. The Rosseta 2 (DE3) pLysS strains harboring pET28a-based plasmids were cultivated at 37ºC and 50 rpm in triplicate. Average doubling times (DT) of the strains were calculated in early exponential phase of growth (OD_{660} = 0.2-0.9).

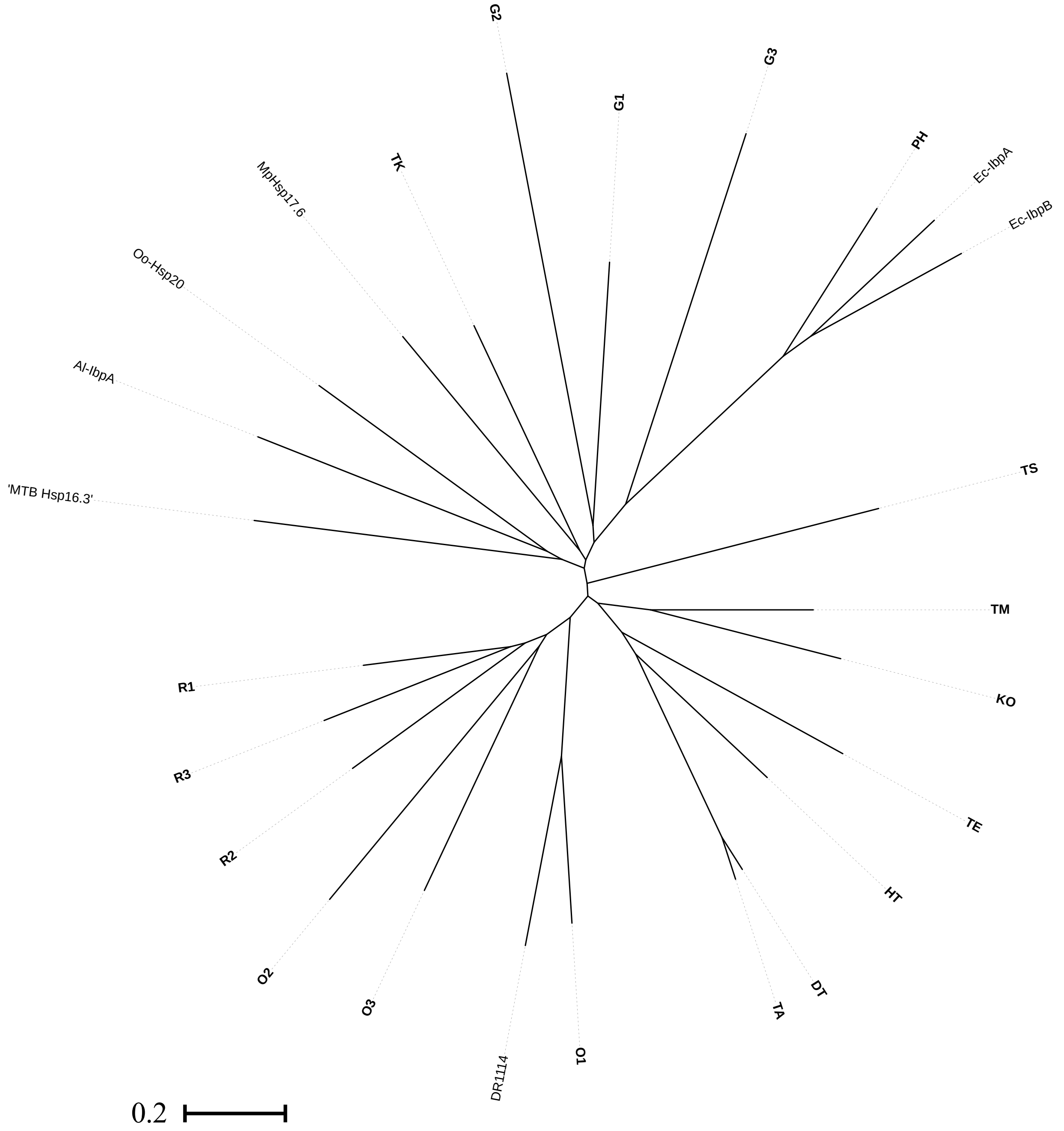

Online Resource 4 Phylogenetic tree of bacterial HSP20 used in present and previous studies.

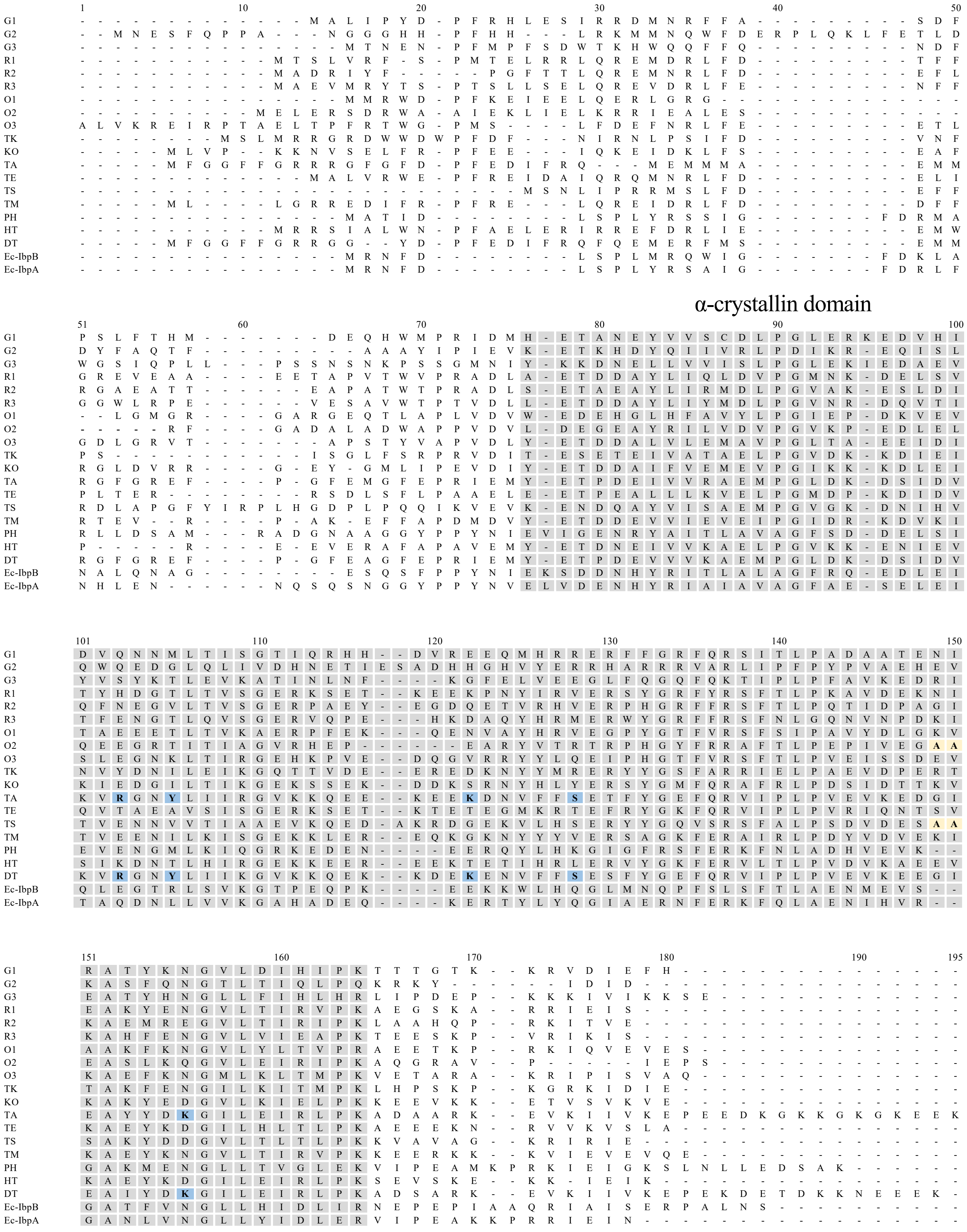

Online Resource 5 Alignment result using amino acid sequences of bacterial HSP20 used in this study. Each color highlighted specific regions as follows; gray, α-crystallin domain; yellow, amino acid residue found only in HSP20s derived from *Oceanithermus* (O2) and *Tepidimonas* (TS) that enhanced multiple stress resistance of *E. coli*; blue, amino acid residue unique to HSP20s derived from *Thermovibrio* (TA) and *Desulfurobacterium* (DT) that did not improve cell viability of *E. coli.*

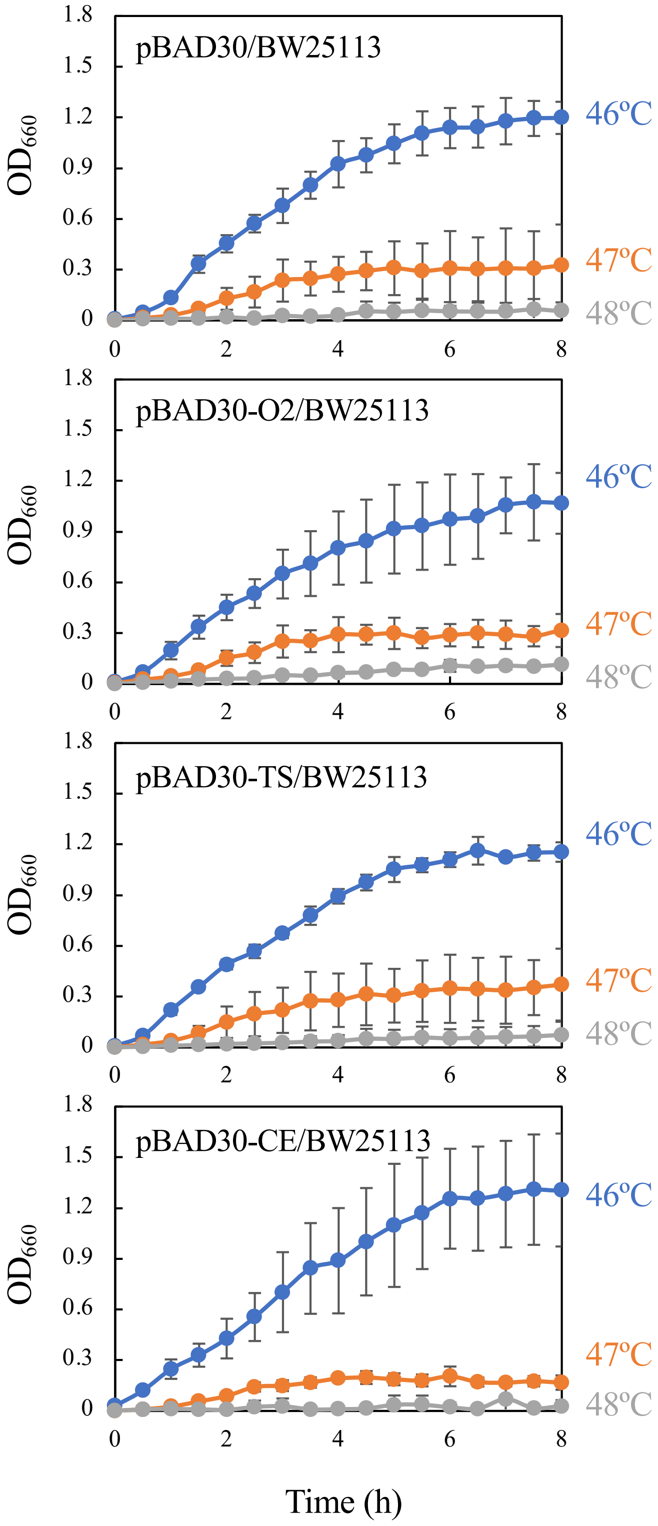

Online Resource 6 Growth curves of pBAD30-expression strains at elevated temperatures.
